# Prevalence of musculoskeletal disorders among Polish white-collar workers: the role of physical activity and risk factors

**DOI:** 10.3389/fpubh.2025.1551728

**Published:** 2025-04-07

**Authors:** Małgorzata Grabara

**Affiliations:** Institute of Sport Science, Jerzy Kukuczka Academy of Physical Education in Katowice, Katowice, Poland

**Keywords:** prolonged sitting, BMI, age, lower back pain, neck pain, sleep duration

## Abstract

**Objective:**

This study evaluated the prevalence of self-reported musculoskeletal disorders (MSDs) among white-collar workers and investigated their associations with physical activity (PA) levels, time spent sitting, sleep duration, and BMI.

**Methods:**

The study included 440 white-collar workers (355 female and 85 male) aged 20–64 years, from randomly selected public institutions, companies, and corporations in the Upper Silesia region, Poland. Participants completed the Nordic Musculoskeletal Questionnaire and the Seven-Day Physical Activity Recall. Statistical analysis included comparisons of PA between workers with and without MSDs and between genders, correlations between MSDs, PA, and sleep duration, and logistic regression assessing associations between potential predictors and MSD presence. Independent variables included age, BMI, daily and occupational sitting time, sleep duration, and PA.

**Results:**

The 12-month prevalence of MSDs was highest for lower back pain (65.9%), neck pain (59.8%), and upper back pain (43.9%), with a similar pattern observed over 7 days. Workers reporting neck pain over the past 12 months engaged in significantly less vigorous PA and high vigorous PA compared to those without neck pain (*p* = 0.019, *r* = 0.11), while those with knee pain reported higher levels of moderate PA (*p* = 0.018, *r* = 0.11). Age and BMI significantly influenced pain in multiple regions, including the neck, shoulder, back, hip/thigh, knee, and ankle/foot. Daily sitting time was a significant predictor for most MSDs, except for hip pain, while sitting during work specifically predicted lower back pain over 12 months. Each additional year of age, unit increase in BMI, and hour of sitting increased the likelihood of pain. Sleep duration and PA levels were not significant predictors.

**Conclusion:**

These findings emphasize the importance of BMI management and reducing prolonged sitting as key workplace interventions, particularly in sedentary occupations.

## 1 Background

Musculoskeletal disorders (MSDs) are a significant health concern, encompassing injuries or dysfunctions that affect various structures within the musculoskeletal system, including muscles, joints, tendons, spinal disks, cartilages, bones, and peripheral nerves (1). Typical symptoms of MSDs include pain, aching, joint stiffness, soreness, tingling, or numbness in muscles, as well as reduced mobility and functional impairment (2–5). These disorders are often exacerbated by prolonged static posture and insufficient or excessive physical activity, ultimately contributing to a reduced quality of life (6).

The prevalence and anatomical distribution of MSDs are often influenced by the specific demands and nature of one’s profession (7). Work-related MSDs are prevalent health issues. Key physical factors contributing to these MSDs include repeated or sustained exertion, excessive force, awkward or extreme postures, and prolonged sitting or standing (1, 8). These disorders are linked to both physical and mental health problems, chronic pain, and disability, resulting in decreased productivity and significant economic burdens (8, 9). MSDs are also associated with increased absenteeism, sick leave, and early retirement (5, 10). In Poland, MSDs rank as the third leading cause of work incapacity, according to the Social Insurance Institution (ZUS) (11).

Recent technological advancements, particularly in office settings, have shifted daily activities toward more sedentary, computer-based tasks, contributing to an increased prevalence of MSDs across various populations (12, 13). This shift from active to predominantly static tasks has introduced new physical and psychosocial risk factors, making MSDs a significant concern in modern lifestyles (13). Although white-collar workers are not among the highest-risk occupational groups for MSDs (2), prolonged sitting, the use of non-ergonomic desks and chairs, physical inactivity, and occupational stress can contribute to the development of MSDs in this group of employees (14). Previous studies have shown that white-collar workers frequently experience musculoskeletal issues, particularly in the lower back, neck, and shoulder, which have significant personal and socio-economic implications. Prolonged sitting has been linked to cognitive impairment, fragmented daytime sleep, and an increased risk of psychological and physiological conditions, including musculoskeletal discomfort (15, 16).

Physical activity (PA), particularly moderate-intensity PA during leisure time, has been shown to prevent and reduce the occurrence of specific MSDs and is associated with a lower risk of long-term sickness absence (15, 17–20). Additionally, adhering to PA guidelines has been linked to a 38% lower risk of emotional exhaustion, while those exceeding the recommended levels experienced an even greater reduction of 47% (21). Engaging in regular PA also contributes to enhanced work ability, supporting both physical and mental resilience in occupational environments (22, 23). Furthermore, the implementation of workplace-based exercises programs, such as resistance and stretching training, has been shown to have a preventive effect on upper extremity MSDs (24).

In addition to insufficient or excessive PA and prolonged sitting time, previous studies have identified various other factors influencing MSDs occurrence. For instance, Walsh et al. (25) reported that individuals with higher body fat were more likely to experience lower back and knee pain. Other recognized risk factors include reduced sleep duration, poor sleep quality, female gender, and older age (26, 27). According to a report by the European Agency for Safety and Health at Work, MSDs were more prevalent in women than men in 2015, and their prevalence increased almost linearly with age—from 49% in individuals under 35 years to 68% in those over 50 years (12).

Although numerous studies have investigated the prevalence of MSDs, few have comprehensively examined a broad range of influencing factors. Research exploring PA and its relationship with MSDs has primarily focused on whether individuals meet or do not meet PA recommendations, rather than analyzing specific PA levels in relation to the occurrence of different MSDs. Furthermore, other potential contributors, such as time spent sitting or sleep duration, are often overlooked. Moreover, there is no consensus on how different PA intensities impact the occurrence of specific MSDs. For example, Sitthipornvorakul et al. (20) indicated in their systematic review that the limited number of studies and their heterogeneity provide insufficient evidence for a clear link between leisure-time PA and neck pain in the working population.

By simultaneously examining multiple risk factors, the present study aims to provide a comprehensive understanding of the determinants of MSDs among white-collar workers. This study aims to assess the prevalence of self-reported MSDs across all body regions, examine gender differences in MSDs occurrence, PA, sitting time, and sleep duration among white-collar workers, and investigate the associations between MSDs and age, PA levels, sitting time, sleep duration, and BMI.

## 2 Methods

### 2.1 The design and setting of the study

This observational, descriptive, cross-sectional study was conducted in randomly selected public institutions, corporations, and companies in the Upper Silesia region of Poland, involving white-collar workers in sedentary jobs.

From a registry of companies in the Silesian Voivodeship operating in information technology, research, and development, four companies were randomly selected. Additionally, two corporations from the financial sector (banks headquartered and/or with branches in the Silesian Voivodeship) were chosen. Furthermore, two institutions related to finance and social insurance, such as the Polish Social Insurance Institution (ZUS), Polish Agricultural Social Insurance Fund (KRUS), or Polish National Health Fund (NFZ), were randomly selected.

After completing the selection process, personal contact was established with the selected companies, corporations, and institutions to request permission to conduct the study. Upon obtaining approval, the research was conducted on-site at participants’ workplaces using a survey-based approach. Before data collection, the study’s purpose was explained to participants, and they were informed about the inclusion criteria. Only individuals who met the criteria and provided informed consent received the questionnaire. Participants completed the survey in the presence of an administrator, who was available to clarify any doubts. Those who did not consent or did not meet the inclusion/exclusion criteria were not provided with the questionnaire.

### 2.2 Participants

The required sample size for this investigation was determined using G*Power software (version 3.1.9.7), developed by Heinrich Heine University in Düsseldorf, Germany. The parameters for the computation included an expected effect size of 0.2, three degrees of freedom (*df*), an alpha error probability of 0.05, and a power of 0.95. Based on these parameters, with a critical chi-squared value of 7.81, a total sample size of 430 participants was determined to be necessary.

The study included 440 white-collar workers aged 20–64 years from public institutions, companies, and corporations in the Upper Silesia region of Poland. The basic characteristics of the participants are presented in Table 1.

**Table 1:** Demographic and anthropometric characteristics of male (M) and female (F) workers.

Variables	F (*n* = 355)	M (*n* = 85)
	Mean ± sd	Mean ± sd
Age [years]	39.68 ± 10.17	38.68 ± 10.25
Body height [cm]	166.28 ± 6.15	179.67 ± 7.96
Body mass [kg]	65.10 ± 11.63	85.48 ± 13.34
BMI [kg/m^2^]	23.55 ± 4.09	26.40 ± 3.24

Age groups	All participants (*n* = 440)	
	*n*	%
20–29 years	87	19.8
30–39 years	149	33.9
40–49 years	116	26.4
50–59 years	78	17.7
>60 years	10	2.3

The inclusion criteria were: (1) full-time employment for at least 1 year, (2) age between 20 and 65 years, (3) a sedentary job, and (4) voluntary consent to participate. The exclusion criteria were: (1) any disease limiting participation in PA, (2) injuries sustained within the past year that limited participation in PA, and (3) pregnancy.

The study was approved by the Bioethics Committee of the Jerzy Kukuczka Academy of Physical Education in Katowice (no. 2/2012) and conformed to the standards established by the Declaration of Helsinki. All participants were informed about the nature and purpose of the study and provided informed consent prior to participation.

### 2.3 Methods and procedures

Anthropometric parameters included body height (BH) and body mass (BM), which were self-reported by participants. The body mass index (BMI) was calculated using BH and BM. Standard BMI ranges were used to classify participants as underweight (<18.5), normal weight (18.5 – <25), overweight (25 – <30), and obesity (≥30) (28).

All participants completed the Nordic Musculoskeletal Questionnaire (NMQ) and the Seven-Day Physical Activity Recall (SDPAR).

The NMQ was used to evaluate musculoskeletal symptoms and disorders (MSDs), including discomfort, numbness, and pain, across nine body regions: neck, shoulder, upper back, elbow, low back, wrist/hand, hip/thigh, knee and ankle/foot (29). Participants reported the occurrence of symptoms within the past 12 months and the past 7 days. To further quantify pain intensity, the NMQ was supplemented with a visual analog scale (VAS), which allowed participants to rate their pain over the past 7 days on a numerical scale from 1 to 10, where 1 represented minimal pain and 10 indicated severe or unbearable pain (30). The NMQ was selected due to its standardization, reliability, and widespread application in epidemiological studies on MSDs. Its structure enables rapid identification of symptoms in nine body regions, making it particularly useful in research involving large populations. Moreover, the NMQ has been translated and adapted into multiple languages, allowing for its use across different countries and cultures, thereby ensuring comparability of results between studies (31, 32).

The PA levels were assessed using the Stanford Seven-Day Physical Activity Recall (SDPAR), a validated tool for measuring PA in healthy adults (33). Participants were asked to estimate the duration (in minutes) of moderate-intensity PA (MPA, 3.0–5.0 METs), vigorous-intensity PA (VPA, 5.1–6.9 METs), and high vigorous-intensity PA (HVPA ≥7.0 METs) (34) for each day of the past week, considering only activities that lasted at least 10 min. Additionally, participants reported the exact number of hours they slept each night during the past week. They were also asked to provide an estimate of their time spent sitting both at work and throughout the day. Participants selected a category that best described their average daily sitting time during the past week: below 2 h, 2–4 h, 4–6 h, 6–8 h, or more than 8 h. To convert the reported PA duration into MET-minutes, the following values were applied: 4 METs for MPA, 6 METs for VPA, and 10 METs for HVPA (34). Based on these calculations, the total energy expenditure from PA was determined per day and per week and expressed in kilocalories (kcal/day, kcal/week) (33–36).

### 2.4 Statistical analysis

The results are presented as means and standard deviations (M ± SD), or as percentages (%). The normality of distribution was tested using the Shapiro–Wilk test.

Between-group differences were analyzed to compare women and men, as well as participants with and without specific MSDs, in terms of quantitative variables. Depending on the data distribution, either the independent *t-*test (for normally distributed data) or the Mann–Whitney *U* test (for non-normally distributed data) was used. Categorical variables were analyzed using Pearson’s Chi-squared test (χ^2^) to compare differences between women and men. The effect size was evaluated using the r index for the Mann–Whitney *U* test, interpreted as follows: 0.1 to <0.3 – small effect, 0.3 to <0.5 – moderate effect, ≥ 0.5 – large effect. For the *t*-test, Cohen’s *d* was used, with the following interpretation: 0.2 to <0.5 – small effect, 0.5 to <0.8 – moderate effect, 0.8 ≤ − strong effect. Cramer’s *V* was applied for Pearson’s Chi-squared test, where 0.1 to <0.2 indicated small effect, 0.2 to <0.4 moderate effect, 0.4 to <0.6 relatively strong effect, 0.6 to <0.8 strong effect, 0.8 to 1 – very strong effect (37).

To examine the influence of BMI category (obesity, overweight, normal weight, and underweight) and gender on the occurrence of specific MSDs, Univariate Analysis of Variance (ANOVA) was conducted. An interaction term between BMI and gender was included to assess potential moderation effects. Effect sizes for ANOVA were estimated using partial eta squared (*η*^2^), interpreted as 0.01 – small effect, 0.06 – medium effect, and 0.14 – large effect (37).

Spearman’s rank correlation analysis was conducted to assess the strength and direction of relationships between the reported number of MSDs, calculated as the sum of complaints over the past 12 months and 7 days, and various levels of PA and sleep duration. The following interpretation of Spearman’s rho (*ρ*) was applied: |ρ| < 0.2 – very weak correlation, 0.2 to <0.4 – weak correlation, 0.4 to <0.6 – moderate correlation, 0.6 to <0.8 – strong correlation, and |ρ| ≥ 0.8 – very strong correlation (37).

The level of significance for all tests was set at *α* = 0.05. Empirical data were analyzed using MS Excel and STATISTICA (version 13.3, TIBCO Software Inc. Palo Alto, CA, United States).

Logistic regression analysis was conducted to assess the associations between independent variables and the presence of various MSDs. The dependent variables were binary, coded as 0 for absence and 1 for presence. Independent variables included in the model were considered potential predictors of MSDs occurrence and comprised age, BMI, daily sitting time, sitting time at work, total sleep time, MPA [MET-min], VPA [MET-min], and TPA [kcal]. Statistical significance was set at *p* < 0.05. The results are presented as odds ratios (OR) with 95% confidence intervals (CI) and corresponding *p*-values. Analyses were conducted using Python with the Pandas, Statsmodels, and scikit-learn libraries.

## 3 Results

### 3.1 Musculoskeletal disorders occurrence and gender-based comparison of study variables in office workers

**Figure 1:**
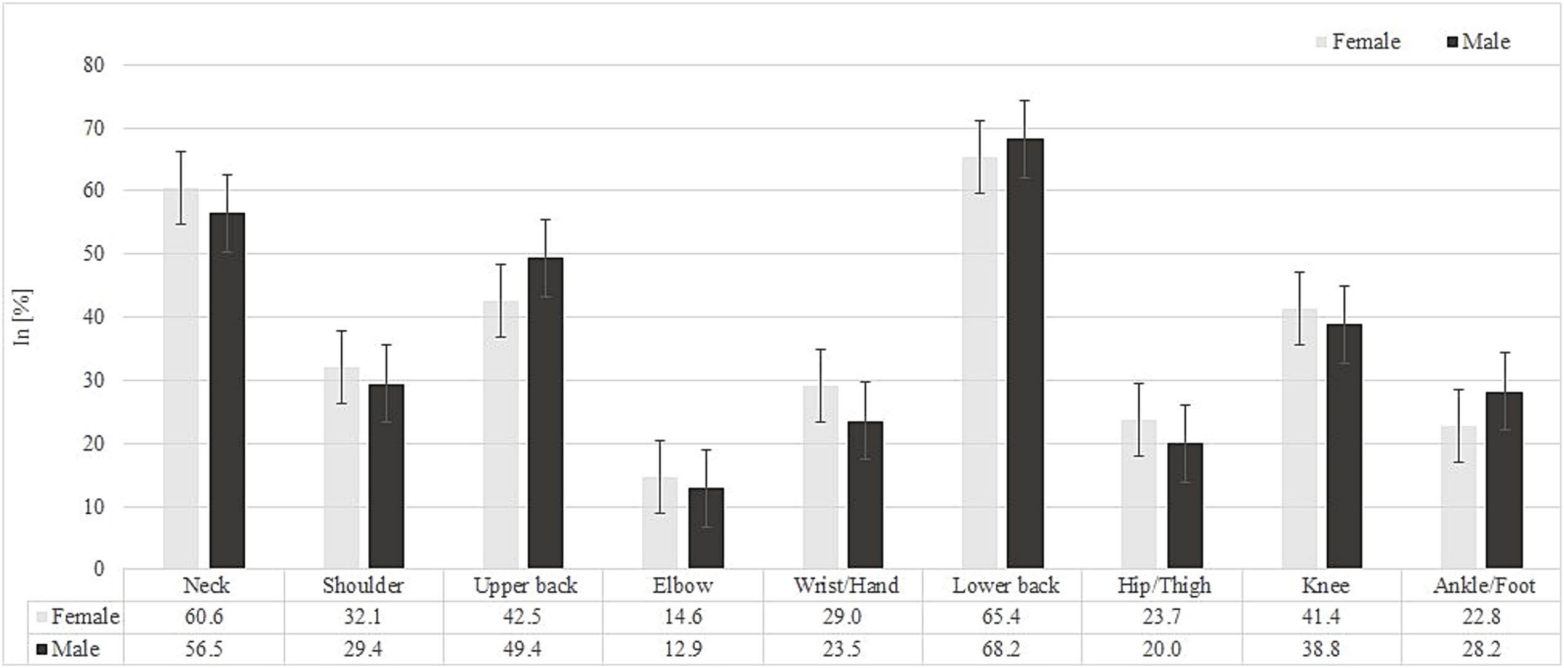
Prevalence of musculoskeletal disorders (MSDs) by body region over the past 12 months among male and female white-collar workers.

**Figure 2:**
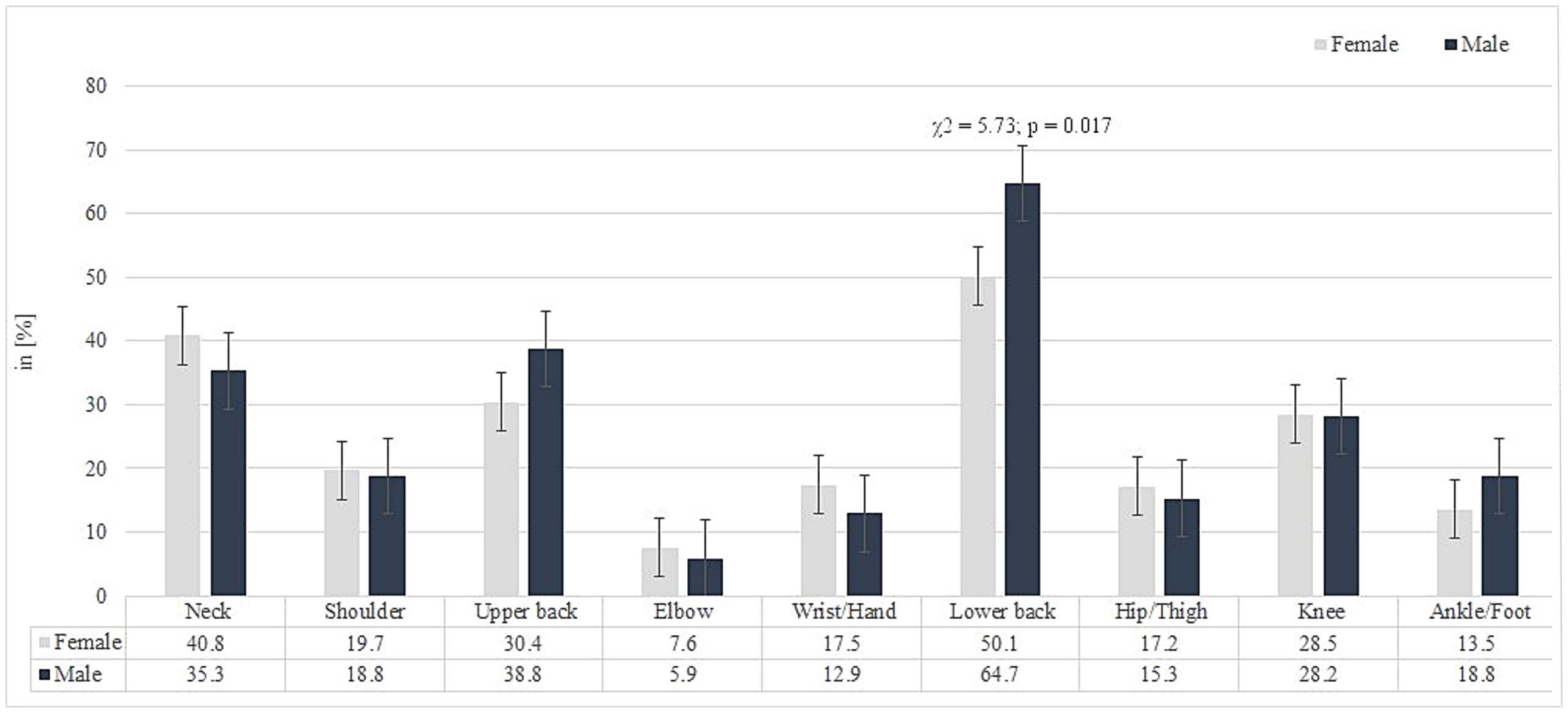
Prevalence of musculoskeletal disorders (MSDs) by body region over the past 7 days among male and female white-collar workers.

The study revealed a 12-month prevalence of MSDs among all participants of 65.9% for lower back pain, 59.8% for neck pain, and 43.9% for upper back pain. The 7-day prevalence was 53% for lower back pain, 39.8% for neck pain, and 32% for upper back pain. The prevalence rates of MSDs over the past 12 months and the past 7 days, separately for women and men, are presented in Figures 1, 2, respectively. A comparative analysis between male and female workers identified a statistically significant difference in the occurrence of low back pain during the past 7 days (*χ*^2^ = 5.73; *p* = 0.017, odds ratio = 1.81, CI [1.11–2.96], *df* = 1). Men were more likely to report low back pain during this period compared to women. The highest average pain intensity was reported for the lower back (4.77 ± 1.89; CI [4.5, 5.0]), followed by knee pain (4.28 ± 2.18; CI [3.90, 4.67]) and neck pain (4.26 ± 1.97; CI [3.96, 4.55]). Significant differences in pain intensity between male and female workers were observed for shoulder pain, with women reporting significantly higher levels of shoulder pain than men (4.46 ± 2.05 vs. 3.00 ± 1.58; *p* = 0.009). The total number of body regions affected reported by all participants over the past 12 months and during the past 7 days was 3.31 ± 2.13 and 2.28 ± 1.83, respectively, with no significant differences observed between male and female workers.

Levels of weekly PA and sleep duration among participants are presented in Table 2. Male workers exhibited higher levels of VPA, HVPA, and TPA compared to female workers (Table 2).

**Table 2:** Weekly physical activity and sleep duration among male and female workers.

Variables	F		M		Sig.
	Mean ± sd	Min–Max	Mean ± sd	Min–Max	
MPA [MET-min]	1,638.85 ± 1,443.0	0–8,000	1,808.24 ± 1,524.36	0–9,800	ns
VPA [MET-min]	792.35 ± 936.04	0–5,640	1,340.47 ± 1,567.66	0–9,900	***p* = 0.002 *r* = 0.15**
HVPA [MET-min]	311.01 ± 811.95	0–8,100	1,095.29 ± 1,642.53	0–9,500	***p* < 0.001 *r* = 0.22**
TPA [kcal]	2,978.76 ± 2,524.39	0–17,838.7	5,972.59 ± 4,860.78	0–35,526.7	***p* < 0.001 *r* = 0.33**
TPA [kcal] mean per day	425.54 ± 360.63	0–2,548.4	853.23 ± 694.4	0–5075.2	***p* < 0.001 *r* = 0.33**
Sleep duration [h]	48.76 ± 4.72	35–60.5	49.48 ± 4.43	40–63	ns

MPA, moderate-intensity physical activity; VPA, vigorous-intensity physical activity; HVPA, high vigorous-intensity physical activity; TPA, total physical activity; F, female workers; M, male workers; p, *p*-value from the Mann–Whitney *U* test; r, effect size; ns, not significant. Significant differences are bolded.

The reported time spent sitting during work and throughout the day is summarized in Table 3. A statistically significant difference in sitting time at work was observed between male and female office workers. Women were more likely to report sitting at work for 4–6 h or 6–8 h, while men more frequently reported sitting for 6–8 h or more than 8 h. Men were also more likely than women to report sitting for 2–4 h. Despite these differences, no statistically significant differences were found between male and female workers in total time spent sitting throughout the day (Table 3).

**Table 3:** Time spent sitting at work and per day among male and female workers.

Time	Time spent sitting at work			Time spent sitting per day		
	F [*n*, %]	M [*n*, %]		F [*n*, %]	M [*n*, %]	
2–4 h	15 (4.2%)	15 (17.6%)	***χ***^***2***^ **= 34.1** ***p* < 0.0001** ***V* = 0.28**	4 (1.1%)	1 (1.2%)	*χ^2^* = 2.93 *p* = 0.402 *V* = 0.08
4–6 h	76 (21.4%)	17 (20%)		36 (10.1%)	9 (10.6%)	
6–8 h	223 (62.8%)	32 (37.6%)		126 (35.5%)	22 (25.9%)	
More than 8 h	41 (11.5%)	21 (24.7%)		189 (53.2%)	53 (62.4%)	

F, female workers; M, male workers; χ^2^, Chi squared test; p, *p*-value; V, Cramer’s *V* (effect size), df = 3. Significant differences are bolded.

### 3.2 Associations between musculoskeletal disorders, physical activity, sleep duration, sitting time, BMI, and age

A comparative analysis of PA between participants who reported and did not report specific MSDs identified a limited number of significant differences. Office workers who reported neck pain over the past 12 months engaged in less VPA and HVPA (179.2 min per week) compared to those without neck pain (220.9 min per week), with a small effect size (*p* = 0.019, r = 0.11). Conversely, participants who reported knee pain over the past 12 months exhibited higher levels of MPA (1776.8 MET-minutes) compared to those without knee pain (1598.7 MET-minutes), also with a small effect size (*p* = 0.018, *r* = 0.11). The presence of specific MSDs in the past 7 days was not associated with significant differences in PA engagement.

**Figure 3:**
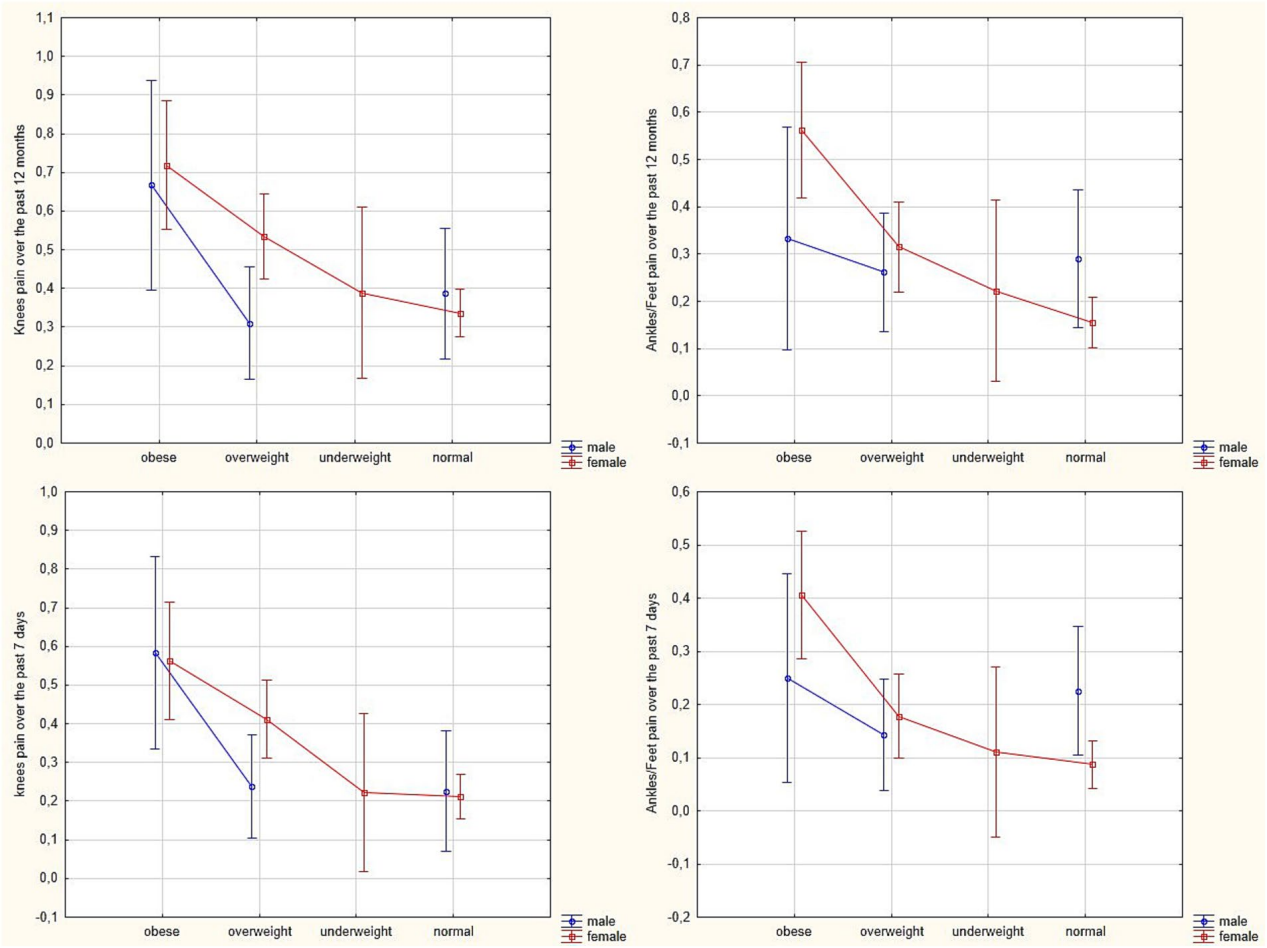
Estimated marginal means of ankle/foot and knee pain occurrence over the past 7 days and the past 12 months among white-collar workers, stratified by BMI category (obese, overweight, underweight, and normal) and gender.

ANOVA revealed a significant main effect of BMI on the frequency of reported knee pain over the past 12 months [*F*(2, 433) = 6.35, *p* = 0.002, *η*^2^ = 0.029] and over the past 7 days [*F*(2, 432) = 8.67, *p* < 0.001, *η*^2^ = 0.039]. The highest frequency of reported pain was observed in the obesity group, followed by the overweight group. However, the interaction between gender and BMI was not statistically significant [*F*(2, 433) = 2.25, *p* = 0.107, *η*^2^ = 0.010] and [*F*(2, 432) = 1.41, *p* = 0.245, *η*^2^ = 0.006], respectively (Figure 3).

A significant main effect of BMI on the frequency of reported ankle and/or foot pain over the past 12 months was also observed [*F*(2, 433) = 3.93, *p* = 0.020, *η*^2^ = 0.018]. The highest frequency of reported pain was observed in the obesity group, followed by the overweight group, while the lowest frequency was found in the underweight group. However, the interaction between gender and BMI was not statistically significant [*F*(2, 433) = 3.01, *p* = 0.051, *η*^2^ = 0.014].

A significant main effect of BMI [*F*(2, 431) = 3.64, *p* = 0.027, *η*^2^ = 0.017] and a significant interaction effect between gender and BMI [*F*(2, 431) = 3.10, *p* = 0.046, *η*^2^ = 0.014] on the frequency of reported ankle and/or foot pain during the past 7 days were also identified. The highest frequency of reported ankle/foot pain was observed in the obesity group, followed by the overweight group (Figure 3).

**Figure 4:**
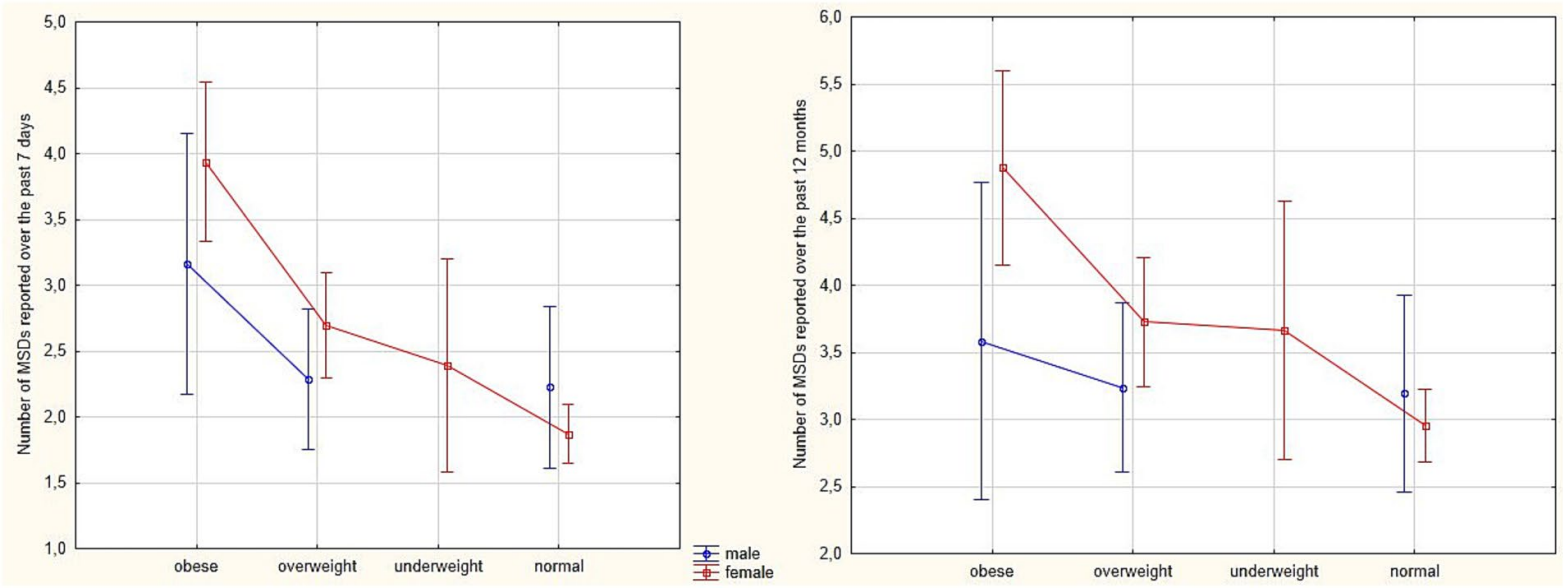
Estimated marginal means of the number of MSDs reported over the past 7 days and the past 12 months among white-collar workers, stratified by BMI category (obese, overweight, underweight, and normal) and gender.

ANOVA also revealed a significant main effect of BMI on the number of MSDs reported over the past 12 months [*F*(2,433) = 4.20, *p* = 0.016, *η*^2^ = 0.019] and over the past 7 days [*F*(2,433) = 9.89, *p* < 0.001, *η*^2^ = 0.044], indicating that the number of reported MSDs was highest in the obese group and decreased with decreasing BMI. However, the interaction between gender and BMI was not statistically significant in either time frame [*F*(2,433) = 2.02, *p* = 0.134, *η*^2^ = 0.009 and *F*(2,433) = 1.99, *p* = 0.137, *η*^2^ = 0.009, respectively], suggesting that the relationship between BMI and MSDs occurrence was similar in men and women (Figure 4).

The Spearman correlation analysis was conducted to examine the relationship between the reported number of MSDs, measured as the sum of complaints over 12 months and 7 days, and various levels of PA and sleep duration. The results revealed no statistically significant correlations between MSDs occurrence over the past 12 months or the past 7 days and PA levels, including MPA, VPA, or HVPA [MET-minutes], as well as TPA [kcal]. However, a very weak negative correlation was observed between the occurrence of MSDs over the last 7 days and time spent sleeping during the week (*ρ* = −0.12, *p* = 0.012), suggesting that higher sleep duration may be associated with a slight reduction in reported MSDs during this period.

The logistic regression analysis for MSDs occurrence over the past 12 months identified age as a significant predictor for pain in the neck, shoulder, upper and lower back, hip and/or thigh, knee, and ankle and/or foot, with each additional year increasing the odds of experiencing pain by 5 to 6%. Similarly, BMI was a significant predictor for these MSDs, with each unit increase in BMI associated with a 13 to 14% increase in the odds of pain. Total daily sitting time was identified as a significant predictor for pain in the neck, shoulder, upper back, knee, and ankle or foot, with each additional hour increasing the odds by 12 to 14%. However, the lower back was found to be the most vulnerable area associated with prolonged sitting throughout the day, with each additional hour increasing the odds of pain by 21%. Time spent sitting at work emerged as a significant variable only for lower back pain, with each additional hour increasing the odds by 15% (Table 4).

**Table 4:** Logistic regression models for specific MSDs over the past 12 months as dependent variables.

Independent variables in model	Dependent variables						
	Neck	Shoulder	Upper back	Lower back	Hip/thigh	Knee	Ankle/foot
Age	*B* = 0.045 ***p* = 0.020** OR 1.05 CI [1.01, 1.09]	*B* = 0.047 ***p* = 0.018** OR 1.05 CI [1.01, 1.09]	*B* = 0.049 ***p* = 0.017** OR 1.05 CI [1.02, 1.08]	*B* = 0.052 ***p* = 0.019** OR 1.05 CI [1.02, 1.08]	*B* = 0.062 ***p* = 0.013** OR 1.06 CI [1.02, 1.10]	*B* = 0.048 ***p* = 0.019** OR 1.05 CI [1.02, 1.08]	*B* = 0.052 ***p* = 0.017** OR 1.05 CI [1.02, 1.09]
Time spent sitting at work	*B* = 0.089 *p* = 0.086 OR 1.09 CI [0.92, 1.30]	*B* = 0.075 *p* = 0.091 OR 1.08 CI [0.91, 1.29]	*B* = 0.083 *p* = 0.078 OR 1.09 CI [0.94, 1.26]	*B* = 0.141 ***p* = 0.024** OR 1.15 CI [1.02, 1.30]	*B* = 0.048 *p* = 0.089 OR 1.05 CI [0.99, 1.12]	*B* = 0.091 *p* = 0.085 OR 1.10 CI [0.95, 1.29]	*B* = 0.91 *p* = 0.082 OR 1.10 CI [0.94, 1.30]
Time spent sitting per day	*B* = 0.132 ***p* = 0.035** OR 1.14 CI [1.02, 1.30]	*B* = 0.116 ***p* = 0.032** OR 1.12 CI [1.01, 1.24]	*B* = 0.121 ***p* = 0.031** OR 1.13 CI [1.01, 1.26]	*B* = 0.191 ***p* = 0.014** OR 1.21 CI [1.05, 1.40]	*B* = 0.048 *p* = 0.364 OR 1.02 CI [0.98, 1.06]	*B* = 0.134 ***p* = 0.031** OR 1.14 CI [1.01, 1.27]	*B* = 0.132 ***p* = 0.034** OR 1.14 CI [1.01, 1.27]
BMI	*B* = 0.125 ***p* = 0.003** OR 1.13 CI [1.05, 1.22]	*B* = 0.132 ***p* = 0.003** OR 1.14 CI [1.06, 1.23]	*B* = 0134 ***p* = 0.004** OR 1.14 CI [1.06, 1.23]	*B* = 0.131 ***p* = 0.036** OR 1.14 CI [1.06, 1.21]	*B* = 0.138 ***p* = 0.005** OR 1.15 CI [1.07, 1.24]	*B* = 0.126 ***p* = 0.004** OR 1.13 CI [1.04, 1.22]	*B* = 0.138 ***p* = 0.005** OR 1.15 CI [1.05, 1.25]
TPA [kcal]; MPA [MET-min]; VPA [MET-min]; Sleep time – not significant with *p* > 0.05.							

TPA, total physical activity; MPA, moderate-intensity physical activity; VPA, vigorous-intensity physical activity; B, coefficient; p, *p*-value; OR, odds ratio; CI, 95% confidence interval. Significant differences are bolded.

A similar pattern was observed for MSDs occurrence over the past 7 days. Each additional year of age increased the odds of pain in the same body areas by 4–6%, while each unit increase in BMI raised the odds by 13–14%. Each additional hour of daily sitting increased the odds of pain in the shoulder, upper back, lower back, knee, ankle and/or foot by 12–15%, with the neck being the most affected (18% increase per hour). The other independent variables were not significant predictors for MSDs (Table 5).

**Table 5:** Logistic regression models for specific MSDs during the past 7 days as dependent variables.

Independent variables in model	Dependent variables						
	Neck	Shoulder	Upper back	Lower back	Hip/thigh	Knee	Ankle/foot
Age	*B* = 0.043 ***p* = 0.018** OR 1.04 CI [1.01, 1.08]	*B* = 0.051 ***p* = 0.015** OR 1.05 CI [1.02, 1.09]	*B* = 0.052 ***p* = 0.016** OR 1.05 CI [1.02, 1.09]	*B* = 0.050 ***p* = 0.021** OR 1.05 CI [1.01, 1.08]	*B* = 0.059 ***p* = 0.017** OR 1.06 CI [1.01, 1.09]	*B* = 0.046 ***p* = 0.022** OR 1.05 CI [1.02, 1.09]	*B* = 0.048 ***p* = 0.021** OR 1.05 CI [1.01, 1.09]
Time spent sitting at work	*B* = 0.101 *p* = 0.079 OR 1.11 CI [0.94, 1.30]	*B* = 0.080 *p* = 0.092 OR 1.08 CI [0.92, 1.30]	*B* = 0.077 p = 0.085 OR 1.08 CI [0.92, 1.26]	*B* = 0.092 *p* = 0.081 OR 1.10 CI [0.93, 1.28]	*B* = 0.044 *p* = 0.098 OR 1.05 CI [0.96, 1.12]	*B* = 0.089 *p* = 0.083 OR 1.09 CI [0.93, 1.27]	*B* = 0.090 *p* = 0.078 OR 1.09 CI [0.94, 1.28]
Time spent sitting per day	*B* = 0.162 ***p* = 0.031** OR 1.18 CI [1.02, 1.36]	*B* = 0.128 ***p* = 0.032** OR 1.14 CI [1.01, 1.28]	*B* = 0.115 ***p* = 0.038** OR 1.12 CI [1.00, 1.26]	*B* = 0.142 ***p* = 0.029** OR 1.15 CI [1.02, 1.31]	*B* = 0.025 *p* = 0.319 OR 1.03 CI [0.97, 1.07]	*B* = 0.129 ***p* = 0.034** OR 1.14 CI [1.01, 1.28]	*B* = 0.128 ***p* = 0.029** OR 1.14 CI [1.02, 1.27]
BMI	*B* = 0.127 ***p* = 0.002** OR 1.14 CI [1.06, 1.23]	*B* = 0.135 ***p* = 0.004** OR 1.14 CI [1.06, 1.24]	*B* = 0.132 ***p* = 0.004** OR 1.14 CI [1.05, 1.23]	*B* = 0.137 ***p* = 0.003** OR 1.15 CI [1.07, 1.23]	*B* = 0.140 ***p* = 0.004** OR 1.15 CI [1.07, 1.24]	*B* = 0.132 ***p* = 0.005** OR 1.14 CI [1.05, 1.22]	*B* = 0.137 ***p* = 0.004** OR 1.15 CI [1.06, 1.25]
TPA [kcal]; MPA [MET-min]; VPA [MET-min]; Sleep time – not significant with *p* > 0.05							

TPA, total physical activity; MPA, moderate-intensity physical activity; VPA, vigorous-intensity physical activity; B, coefficient; p, *p*-value; OR, odds ratio; CI, 95% confidence interval. Significant differences are bolded.

The models also included time spent sleeping, MPA, VPA, and total energy expenditure from PA, but none of these variables were statistically significant for any MSDs. No significant variables were identified for wrist and/or hand, or elbow pain over the past 12 months and the past 7 days.

## 4 Discussion

The study demonstrated a high prevalence of MSDs among the entire group of white-collar workers over the past 12 months, with 66% reporting lower back pain, 60% neck pain, 44% upper back pain, 41% knee pain, 32% shoulder pain, 28% wrist/hand pain, 24% ankle/foot pain, 23% hip/thigh pain, and 14% elbow pain. The 7-day prevalence rates were lower but followed a similar distribution to the 12-month prevalence rates. Janwantanakul et al. (16) identified the neck (42%), lower back (34%), upper back (28%), and wrist/hand (20%) as the most prevalent MSDs attributed to work among office workers. They also observed that female office workers were more likely to report MSDs in the neck, shoulder, upper back and ankle/foot areas compared to male workers (16). Similar findings regarding the more frequent occurrence of MSDs among female workers compared to male workers have been reported in other studies (13, 38, 39). In contrast, the present study revealed that male workers were more likely to report lower back pain than female workers (65% vs. 50%). This result may be explained by the fact that men might be less likely to adhere to preventive measures for MSDs, including ergonomic principles (40). Additionally, psychosocial factors related to the workplace, such as workload and job control, may contribute to the development of chronic low back pain. Men may experience greater social pressure associated with professional roles, leading to increased stress and muscle tension, which in turn may exacerbate back pain (41, 42). This result aligns with the findings of Kibret et al. (42), who observed that male bank workers were more frequently affected by work-related MSDs (WMSDs) than their female counterparts. Similarly, Grabowska and Kwaśniewska (40) reported that lower back pain was the most prevalent MSD, followed by neck and upper back pain, with women more frequently experiencing neck pain and men more frequently reporting lower back pain, which is in line with the present study. Akrouf et al. (39) found that among 750 bank workers, the most commonly affected body regions were the neck (54%), lower back (51%), shoulder (49%), and upper back (38%). Another study involving 300 bank workers reported that the annual prevalence of musculoskeletal discomfort was highest in the lower back (52%), followed by the neck (48%), shoulder (40%), and upper back (39%) (38). In contrast, the findings of the present study indicate a lower prevalence of shoulder pain among white-collar workers compared to these previous studies, but a higher prevalence of knee pain. Certain discrepancies between the findings of this study and those cited above may be attributed to geographical differences, as the studies were conducted in various regions of the world. These differences may reflect variations in working conditions, including ergonomic standards, workplace culture, job-related stress, and the level of occupational physical strain. Such factors can vary across countries, potentially influencing the prevalence and reporting of MSDs. A study conducted in Poland by Zejda et al. (43) found that office workers most frequently reported MSDs affecting the neck (56%), lower back (50%), upper back (50%), wrist/hand (30%), shoulder (27%), and elbow (13%). Based on a systematic review by Demissie et al. (13), 15 out of 25 studies identified the lower back, neck, upper back, and shoulders as the most affected body parts among office workers using computers. These findings highlight differences in the prevalence and distribution of MSDs among various groups of white-collar workers, with lower back and neck pain consistently emerging as the most common complaints in this occupational group, as also observed in the present study.

This study found that male workers exhibited higher levels of VPA, HVPA, and TPA compared to female workers, aligning with findings from previous studies (44–46). Interestingly, PA levels demonstrated contrasting patterns in relation to different MSDs. Participants experiencing neck pain engaged in lower levels of VPA and HVPA than those without neck pain, suggesting that neck discomfort may act as a barrier to participation in VPA. A somewhat different finding was observed in a study conducted among Swedish adults, which revealed that engaging in moderate-intensity aerobic PA for more than 60 min per week was associated with a reduced risk of experiencing neck and shoulder pain (47). Similarly, a study among Italian office workers found that individuals who exceeded PA recommendations reported lower prevalence of neck/shoulders, arms and lower back pain compared to those who did not meet these recommendations (21). However, the authors did not provide a detailed analysis of weekly PA volume in relation to specific MSDs. The present study also found that participants with knee pain reported higher levels of MPA than those without knee pain. This finding suggests a potential association between increased MPA and strain in this region, which contrasts with previous studies (18, 48). While Haljaste and Unt (48) observed that prior participation in endurance sports was associated with a higher risk of knees problems, their study focused on former athletes, making direct comparisons challenging. Similarly, Pan et al. (18) found that knee and hip pain was strongly associated with PA levels. Specifically, knee pain showed a negative correlation with VPA, suggesting that such pain may limit engagement in VPA, whereas hip pain was associated with reduced MPA and increased low-intensity PA (LPA) (18). Hildebrandt et al. (17) reported that workers with sedentary jobs who did not engage in sports were more likely to experience symptoms of lower back pain compared to non-sedentary workers. In contrast, the present study did not identify significant associations between lower back pain and PA levels, differing from findings in prior research findings. Furthermore, no relationships were observed between the number of reported MSDs over the past 12 months or the last 7 days and various PA levels. This finding is inconsistent with previous research. For example, Grabara (46) reported that individuals experiencing a higher incidence of MSDs were less likely to engage in VPA and TPA. Similarly, Pan et al. (18) found that musculoskeletal pain at multiple sites was consistently associated with increased low-intensity PA and reduced engagement in MPA and VPA. An appropriately designed exercise program may help reduce fear-avoidance behavior, which often leads to decreased PA and worsened MSDs, and facilitate functional improvements, even in the presence of ongoing pain (43). Implementing structured PA interventions may not only mitigate the impact of MSDs on daily functioning but also counteract the potential biases introduced by self-reported pain and activity levels.

The discrepancies between these findings may be attributed to differences in study populations and measurement tools used to assess PA and MSDs. Additionally, the self-reported nature of PA and MSDs in many studies, including this one, may introduce recall bias and affect the accuracy of observed associations.

In addition, the current study identified a very weak negative correlation between the number of MSDs reported over the last 7 days and the time spent sleeping during the week. This observation suggests that longer sleep duration may be modestly associated with a reduction in reported MSDs during this period. Although the correlation is weak, it aligns with evidence suggesting that adequate sleep supports recovery and reduces musculoskeletal complaints. For instance, a previous study involving nursing professionals demonstrated that poor sleep quality significantly increased the risk of developing MSDs (49).

The study found that obese and overweight individuals reported knee pain significantly more often than those with normal weight or underweight, suggesting that BMI is a significant predictor of knee pain, with no BMI-dependent gender effect observed. Similarly, ankle and/or foot pain was reported more frequently in obese and overweight individuals, confirming the role of excess weight in these MSDs. Additionally, a BMI-gender interaction was observed for ankle/foot pain over the past 7 days, indicating that the relationship between BMI and pain frequency differed by gender. Women consistently reported more ankle and/or foot pain than men, particularly in the underweight and normal BMI groups. However, in the obese and overweight groups, this gender difference diminished, and in some cases, men reported slightly more ankles/feet pain than women. The findings also indicate that BMI is a significant risk factor of MSD occurrence, with being obese or overweight associated with a greater number of reported MSDs. However, the interaction between gender and BMI was not significant, suggesting that the relationship between BMI and MSDs remained consistent across men and women. This analysis, which considers BMI classification and gender, does not indicate a higher prevalence of other MSDs but highlights those likely associated with excessive body weight, such as knee and ankle/foot pain. It also confirms that a higher BMI is linked to a greater number of MSDs. Therefore, reducing body weight may be a key strategy in decreasing the incidence of MSDs and alleviating knees and feet pain.

The analysis of potential predictors influencing for MSDs identified age and BMI as significant risk factors for pain in the neck, shoulder, upper back, lower back, hip and/or thigh, knee, and ankle and/or foot over the past 12 months and 7 days. Time spent sitting per day also emerged as a significant risk factor for these MSDs, except for hip and/or thigh pain. Additionally, sitting time at work was identified as a significant risk factor for lower back pain over the past 12 months. Each additional year of age, each unit increases in BMI, and each additional hour of sitting time increases the likelihood of experiencing pain in these body regions. However, the analysis revealed that, within this dataset, PA levels and sleep duration did not show a direct relationship with the frequency of MSDs.

Biological changes associated with aging, such as degenerative processes in muscles, tendons, ligaments, and joints, contribute to the pathogenesis of MSDs (50). Additionally, prolonged work experience in older individuals may lead to cumulative fatigue and muscle tension, increasing the risk of WMSDs over time (42). A study by Kibret et al. (42) identified age as a significant predictor of WMSDs. They observed that bank workers aged 30–39 years were 5.6 times more likely to experience WMSDs, and those aged above 40 years were 5.7 times more likely to be affected compared to workers aged 20 to 29 years. Similarly, Marzban et al. (51) and Demissie et al. (13), in their systematic review and meta-analysis, also highlighted age as a critical determinant of MSDs, linking older age groups to higher susceptibility. In contrast, Sulaiman et al. (38) did not find a significant association between age and the prevalence of MSDs.

A higher BMI is associated with increased mechanical loads on the spine and lower limb joints, contributing to the development of MSDs. Excess body mass leads to greater muscular fatigue during basic activities like standing or sitting and reduces mechanical efficiency, particularly during movement. Individuals with obesity also experience higher energy expenditure during physical tasks, reduced muscle strength (especially in the lower limbs), and impaired motor function, which can hinder daily activities such as walking or lifting objects (52, 53). BMI has been identified as a significant risk factor for MSDs among office workers, as demonstrated by Noraziera et al. (54). Similarly, Viester et al. (55) reported that a high BMI, indicative of overweight and obesity, was associated with an increased 12-month prevalence of MSDs overall. Specifically, overweight individuals were more likely to report pain in the upper and lower extremities, whereas obesity was more strongly associated with pain in the neck, shoulders, back, and lower extremities (55). Gerovasili et al. (45) examined the associations between PA, BMI, and MSDs in the Norwegian general population and found that obese women and men had a 20% higher risk of chronic back and neck/shoulder pain, while higher levels of PA reduced the risk of these MSDs. However, the study did not assess other MSDs, which limits the comparability of their findings with the present study. Additionally, some studies have not identified BMI as a predictor of specific MSDs, suggesting that other contributing factors, such as occupational stress may play a role (42, 56).

As observed in the present study, time spent sitting emerged as a significant predictor of MSDs. Prolonged sitting places stress on the neck and thoracic extensor muscles due to their constant contraction to maintain head posture, which over time can lead to muscle fatigue and neck pain. Tilting the neck beyond 30° further increases muscle fatigue, as smaller spinal muscles compensate for reduced spinal support, resulting in overload and potential injury (13). Prolonged sitting often leads to flattened lumbar lordosis, placing the lumbar spine in a flexed position compared to standing. Many individuals adopt a slumped sitting posture for extended periods, which increases the load on passive spinal structures, such as ligaments and intervertebral disks, due to insufficient muscular support. As a result, prolonged sitting induces significant compressive loads on the lumbar spine, exceeding those observed in a standing position (57–59). Bontrup et al. (59) reported a modest association between general sitting behavior and the prevalence of chronic LBP and pain-related functional disability among participants. Similarly, Etana et al. (60) found that bank workers who remained in the same position (sitting or standing) for two or more hours had twice the risk of developing WMSDs compared to those who alternated between positions. They also identified awkward postures as a significant risk factor contributing to the occurrence of WMSDs (60). This suggests that prolonged static postures during work may contribute to musculoskeletal strain and disorders. In contrast, Noraziera et al. (54) did not observe an association between time spent sitting and the prevalence of MSDs, which may be attributed to the relatively small number of participants reporting sitting durations below 2 h (one participant) and between 2 and 4 h (17 participants), whereas the majority (68 participants) reported sitting for more than 4 h per day. Additionally, the authors did not include higher sitting time categories in the questionnaire, potentially limiting the ability to capture variations in prolonged sitting duration. Thus, differences in questionnaire design may contribute to discrepancies in research findings.

This study has several limitations that should be considered when interpreting the findings. First, the assessment of PA levels and the occurrence of MSDs was based on subjective and retrospective self-reports, which may introduce recall bias and reduce data accuracy. Second, the number of hours spent working on a computer was not recorded, which may have impacted the prevalence of MSDs, particularly among white-collar workers. Additionally, other potentially relevant factors, such as marital status and the use of substances (e.g., tobacco and alcohol), were not examined. Furthermore, there was a significant gender imbalance in the sample, with a notably higher number of female participants than male participants. This limitation is particularly important, as the study included comparative analyses between genders, which may have been influenced by the unequal group sizes.

## 5 Conclusion

This study demonstrated a high prevalence of MSDs among white-collar workers, with lower back, neck, and upper back pain being the most commonly reported complaints over both the past 12 months and the past 7 days. Men were more likely to report low back pain over the past 7 days, whereas women experienced significantly higher shoulder pain intensity, though no significant differences were found in the total number of affected body regions between genders.

Key risk factors for MSDs included age, BMI, and sitting time. Older individuals and those with a higher BMI were more likely to experience pain across multiple body regions. Prolonged sitting also increased MSD risk, with each additional hour of daily sitting heightening the likelihood of pain in several body areas. These findings highlight the importance of BMI management and reducing prolonged sitting as key workplace interventions, particularly in sedentary occupations.

Despite previous evidence supporting the protective role of PA, this study did not identify PA levels or sleep duration as significant predictors of MSDs. However, a few notable associations were observed — participants with neck pain engaged in less VPA, while those with knee pain reported higher levels of MPA.

## Data Availability

The raw data supporting the conclusions of this article will be made available by the authors, without undue reservation.
